# Obeticholic Acid Protects against Gestational Cholestasis-Induced Fetal Intrauterine Growth Restriction in Mice

**DOI:** 10.1155/2019/7419249

**Published:** 2019-11-15

**Authors:** Wei Chen, Xing-Xing Gao, Li Ma, Zhi-Bing Liu, Li Li, Hua Wang, Lan Gao, De-Xiang Xu, Yuan-Hua Chen

**Affiliations:** ^1^Department of Histology and Embryology, Anhui Medical University, Hefei, China; ^2^Key Laboratory of Environmental Toxicology of Anhui Higher Education Institutes, Anhui Medical University, Hefei, China; ^3^The First Affiliated Hospital, Anhui Medical University, Hefei, China

## Abstract

Gestational cholestasis is a common disease and is associated with adverse pregnancy outcomes. However, there are still no effective treatments. We investigated the effects of obeticholic acid (OCA) on fetal intrauterine growth restriction (IUGR) during 17*α*-ethynylestradiol- (E2-) induced gestational cholestasis in mice. All pregnant mice except controls were subcutaneously injected with E2 (0.625 mg/kg) daily from gestational day (GD) 13 to GD17. Some pregnant mice were orally administered with OCA (5 mg/kg) daily from GD12 to GD17. As expected, OCA activated placental, maternal, and fetal hepatic FXR signaling. Additionally, exposure with E2 during late pregnancy induced cholestasis, whereas OCA alleviated E2-induced cholestasis. Gestational cholestasis caused reduction of fetal weight and crown-rump length and elevated the incidence of IUGR. OCA decreased the incidence of IUGR during cholestasis. Interestingly, OCA attenuated reduction of blood sinusoid area in placental labyrinth layer and inhibited downregulation of placental sodium-coupled neutral amino acid transporter- (SNAT-) 2 during cholestasis. Additional experiment found that OCA attenuated glutathione depletion and lipid peroxidation in placenta and fetal liver and placental protein nitration during cholestasis. Moreover, OCA inhibited the upregulation of placental NADPH oxidase-4 and antioxidant genes during cholestasis. OCA activated antioxidant Nrf2 signaling during cholestasis. Overall, we demonstrated that OCA treatment protected against gestational cholestasis-induced placental dysfunction and IUGR through suppressing placental oxidative stress and maintaining bile acid homeostasis.

## 1. Introduction

Gestational cholestasis, also known as intrahepatic cholestasis of pregnancy (ICP), is defined as the presence of pruritus in combination with elevated levels of serum total bile acid (TBA) and liver transaminases [1]. Gestational cholestasis is one of the most common obstetric diseases and occurs usually in the second half of pregnancy until delivery. The incidence of gestational cholestasis is from 0.4% to 15% in different countries, ethnic populations, and climatic conditions [2, 3]. Several epidemiological studies reported the association between gestational cholestasis and increased risks of pregnancy complications, such as gestational diabetes mellitus, preeclampsia, dyslipidemia, and later liver and biliary tree cancer, suggesting the diverse influence of gestational cholestasis on maternal outcomes [4–7]. On the other hand, numerous studies in humans and rodents demonstrated that gestational cholestasis elevated risks of adverse fetal outcomes, such as intrauterine fetal death, spontaneous preterm delivery, respiratory distress syndrome, and a low (<7) 5-minute Apgar score [8–10]. Moreover, a study in human and rodent animal demonstrated that female offspring from cholestatic mothers developed a severe obese, diabetic phenotype with hepatosteatosis following a Western diet in adult, highlighting that gestational cholestasis can program metabolic disease in the offspring [11]. Fetal intrauterine growth restriction (IUGR), defined as fetus that fails to achieve his growth potential, is highly prevalent obstetric complications and may cause serious adverse health consequences for the baby [12–14]. Nevertheless, the effects of gestational cholestasis on fetal IUGR remain to be explored in mice.

The majority of studies reported that falsely elevated estradiol level during late pregnancy was one of the leading contributors to cause gestational cholestasis [15, 16]. Thus, estradiol treatment during late pregnancy was usually used to induce gestational cholestasis in animals. High estradiol exposure during late pregnancy could dysregulate bile acid homeostasis through inhibiting hepatic farnesoid X receptor (FXR) activity [17]. Ursodeoxycholic acid (UDCA) is a commonly used drug for the treatment of gestational cholestasis in clinical practice. However, several animal experiments and epidemiological studies showed that UDCA did not alleviate gestational cholestasis-induced adverse pregnancy outcomes, such as preterm delivery [18–21]. The limited efficacy of UDCA in various cholestatic conditions urges for development of novel therapeutic approaches. Numerous studies recognized that excess reactive oxygen species (ROS) played an important role in cholestasis-induced liver injury and adverse fetal outcomes [22–24]. Obeticholic acid (OCA) is a pharmaceutical currently in clinical trials to treat nonalcoholic steatohepatitis and is also a synthetic agonist of FXR [25, 26]. Moreover, an earlier research showed that OCA as a potent FXR ligand can protect against E217*α* cholestasis [27]. However, the effects of OCA on E217*α* cholestasis-induced fetal development have not been proved. FXR ligand is a ligand-activated transcription factor that is highly expressed in human and rodent animal placentas [28, 29]. Several studies found that FXR has an antioxidant effect [30, 31]. A recent study showed that OCA pretreatment protects against sepsis-induced acute kidney injury through inhibiting renal oxidative stress in mice [32]. Nevertheless, it is not known whether OCA treatment can alleviate gestational cholestasis-induced fetal IUGR.

The aim of the present study was to investigate the effects of OCA pretreatment on fetal IUGR during 17*α*-ethynylestradiol- (E2-) induced gestational cholestasis in mice. The present study found that gestational cholestasis induced by E2 resulted in fetal death and IUGR in mice. OCA pretreatment activated placental and hepatic FXR signaling. Additionally, OCA pretreatment protected against gestational cholestasis-induced placental dysfunction and fetal IUGR through suppressing placental oxidative stress and maintaining bile acid homeostasis.

## 2. Material and Methods

### 2.1. Chemicals and Reagents

17*α*-Ethynylestradiol (E2) and obeticholic acid (OCA) were purchased from Sigma Chemical Co. (St. Louis, MO). FXR, HO-1, 3-NT, and *β*-actin antibodies were purchased from Santa Cruz Biotechnologies (Santa Cruz, CA). NOX-4, Nrf2, SNAT2, and Lamin A/C antibodies were purchased from Abcam (Cambridge, MA). Keap1 antibody was purchased from Bioss Antibodies (Beijing, China). Chemiluminescence (ECL) detection kit was from Pierce Biotechnology (Rockford, IL). TRI reagent was from Molecular Research Center, Inc. (Cincinnati, Ohio). RNase-free DNase was from Promega Corporation (Madison, WI).

### 2.2. Animals and Treatments

ICR mice (8~10 weeks old) were purchased from Beijing Vital River whose foundation colonies were all introduced from Charles River Laboratories, Inc. Before the experiments, the animals were maintained on standard chow and water ad libitum and housed in a temperature (20–25°C) and humidity (50 ± 5%) controlled room under a 12 h light/dark cycle. For mating purposes, four females were housed overnight with two males starting at 9:00 PM. Females were checked by 7:00 AM the next morning, and the presence of a vaginal plug was designated as gestational day (GD) 0. Forty-eight pregnant mice were randomly divided into four groups on GD12. In the E2 alone and the OCA+E2 groups, pregnant mice were subcutaneously injected with E2 (0.625 mg/kg) daily from GD13 to GD17. In the OCA alone and OCA+E2 groups, pregnant mice were orally administered with OCA (5 mg/kg) daily from GD12 to GD17. The dose of OCA used was chosen on the basis of previous study [26]. All dams were sacrificed on GD18, and gravid uterine weights were recorded. For each litter, the number of live fetuses, dead fetuses, and resorption sites was counted. Live fetuses and placentas were weighed. Maternal serum was collected for measurement of total bile acid (TBA) and liver transaminases. This study was approved by the Association of Laboratory Animal Sciences at Anhui Sciences and the Center for Laboratory Animal Sciences at Anhui Medical University.

### 2.3. Immunohistochemistry (IHC)

The mouse placentas were fixed with 4% formaldehyde and embedded in paraffin. Tissue blocks were cut for 5 *μ*m thick and stained with hematoxylin and eosin (H&E) for histopathological examination. For IHC, placental sections were deparaffinized and rehydrated in a graded ethanol series. After Ag retrieval and quenching of endogenous peroxidase, sections were incubated with anti-FXR, anti-3-NT, anti-Nrf2, and anti-Snat2 mAbs (1 : 200 dilution) at 4°C overnight. The color reaction was developed with HRP-linked polymer detection system (Golden Bridge International, WA, USA) and counterstained with hematoxylin.

### 2.4. Biochemical Analysis

Before slaughtering the maternal mice, collect 1 ml of venous blood in a centrifuge tube, place the collected blood sample in a refrigerator at 4°C for 3 hours, and centrifuge it at 3000×g for 10 minutes for serum separation. Maternal serum total bile acid (TBA), alanine aminotransferase (ALT), and aspartate aminotransferase (AST) levels were detected by using an automated bio analyzer (Dirui CS-T300, Ltd, Changchun, Jilin Province, China).

### 2.5. Determination of Glutathione (GSH) Content

Placental GSH was measured by the method of Griffith [33]. The levels of GSH were expressed as *μ*mol/g tissue.

### 2.6. Determination of Malondialdehyde (MDA) Level

Placental lipid peroxidation was quantified by measuring the MDA level as described previously [34]. MDA level was expressed as nmol/mg tissue.

### 2.7. Total RNA Isolation and Quantitative RT-PCR (qRT-PCR) Assay

Mouse placental tissues were homogenized in 1.2 ml TRIzol reagent (Ambion, USA). Total RNA (1.0 *μ*g) was treated with the RNase-free DNase and reverse-transcribed with AMV (Pregmega). The gene-specific primers are listed in Table 1. The PCR amplification reaction was amplified in 50 cycles in a three-step process of denaturation (95°C for 15 s), annealing (60°C for 15 s), and extension (72°C for 30 s). The relative ratio of the target gene was calculated using a LightCycler 480 SYBR GreenIkit software (Roche Diagnostics).

### 2.8. Immunoblots

For total protein extraction, 70 mg tissue was cut and added to 500 *μ*l RIPA lysate (containing protease inhibitor) to prepare lysate. For nuclear protein extraction, 200 mg was added to the PBS salt solution containing the protease inhibitor three times, and the homogenate was passed through a 200-mesh screen and centrifuged at 1000×g for 5 minutes. The supernatant was discarded and added 1 ml PBS solution, and the resuspended cells were centrifuged at 1000×g for 5 minutes in a centrifuge. After discarding the supernatant, 1 ml of a PBS salt solution containing a protease inhibitor and 0.1% NP-40 was added, and the mixture was repeatedly centrifuged at 10000×g for 20 seconds. Dry the liquid, collect the nuclear, add 100 *μ*l of PBS salt solution containing protease inhibitor to the precipitate, and using an electric microhomogenizer, cleave on ice for 30 minutes. Centrifuge at 12000×g for 15 minutes. After collecting the supernatant, according to the manufacturer's instructions using the BCA protein assay kit (Thermo Scientific Pierce Microplate), quantify protein. In brief, lysates were separated by SDS-PAGE electrophoresis in 10% gels. Total proteins were transferred to a polyvinylidene difluoride membrane (Millipore, Billerica, MA). The membrane was incubated at room temperature for 2 hours using the following specific antibodies: FXR (1 : 1000 dilution), SNAT2 (1 : 1000 dilution), HO-1 (1 : 1000 dilution), NOX-4 (1 : 1000 dilution), 3-NT (1 : 1000 dilution), Nrf2 (1 : 500 dilution), and Keap1 (1 : 200 dilution). Use *β*-actin (1 : 10000 dilution) as total protein load control. Lamin A/C (1 : 2000 dilution) was used as nuclear protein load control. It was subsequently developed using the enhanced chemiluminescent (ECL) assay kit.

### 2.9. Statistical Analysis

Quantified data were expressed as means ± S.E.M. A comparison between the two groups was performed using a *t*-test. Differences between the different groups were determined using the ANOVA and Student-Newman-Keuls post hoc methods. *P* < 0.05 was considered statistically significant.

## 3. Results

### 3.1. OCA Pretreatment Activated FXR Signaling

**Figure 1:**
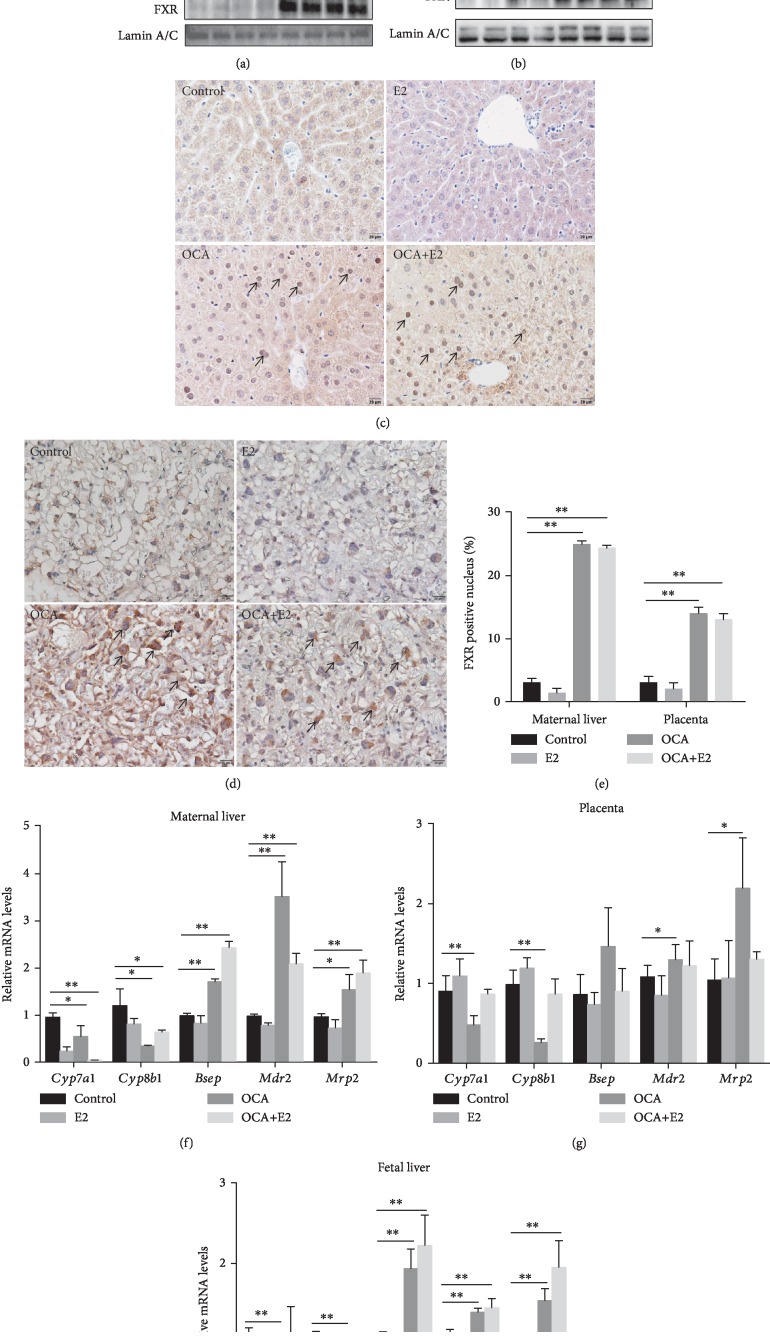
OCA pretreatment activated FXR signaling. All pregnant mice except controls were s.c. injected with E2 (0.625 mg/kg) once daily from GD13 to GD17. In the OCA and OCA+E2 groups, pregnant mice were administered with OCA (5 mg/kg) by gavage once daily from GD12 to GD17. All dams were sacrificed 4 hours after the last administration of OCA. (a, b) Hepatic and placental nuclear FXR were measured using immunoblot. Representative gels were shown. (a) Hepatic nuclear FXR/Lamin A/C. (b) Placental nuclear FXR/Lamin A/C. (c, d) Nuclear translocation of hepatic and placental FXR was analyzed using IHC. (c) Representative photomicrographs were shown. Original magnification: ×400. Nuclear translocation of FXR was observed in hepatocyte (arrows). (d) Representative photomicrographs were shown. Original magnification: ×400. Nuclear translocation of FXR was observed in mononuclear sinusoidal TGCs of the labyrinth zone (arrow). (e) Hepatic and placental FXR-positive cells were analyzed. (f–h) *Cyp7a1*, *Cyp8b1*, *Bsep*, *Mdr2*, and *Mrp2* mRNA were measured using real-time RT-PCR. (f) Relative mRNA levels in maternal liver. (g) Relative mRNA levels in placenta. (h) Relative mRNA levels in fetal liver. Quantified data were expressed as means ± S.E.M. of six samples from six different pregnant mice. ^∗^*P* < 0.05, ^∗∗^*P* < 0.01.

The effects of OCA on FXR signaling in maternal liver were analyzed. The level of maternal hepatic nuclear FXR was markedly elevated in OCA-pretreated mice (Figure 1(a)). The effects of OCA on FXR signaling in placenta were then analyzed. As shown in Figure 1(b), OCA pretreatment increased the nuclear protein level of placental FXR in mice. Immunohistochemistry showed that OCA promoted nuclear translocation of FXR in maternal hepatocytes and mononuclear sinusoidal trophoblast giant cells of the placental labyrinth zone (Figures 1(c)–1(e)). FXR target genes in maternal liver, placenta, and fetal liver were measured using real-time RT-PCR. As shown in Figures 1(f)–1(h), OCA pretreatment upregulated the gene expressions of *Bsep*, *Mdr2*, and *Mrp2*, three target genes of FXR, in maternal liver, placenta, and fetal liver. By contrast, OCA pretreatment reduced the gene expressions of *Cyp7a1* and *Cyp8b1*, two suppressive target genes of FXR, in maternal liver, placenta, and fetal liver (Figures 1(f)–1(h)).

### 3.2. OCA Alleviated 17*α*-Ethynylestradiol-Induced Gestational Cholestasis

**Figure 2:**
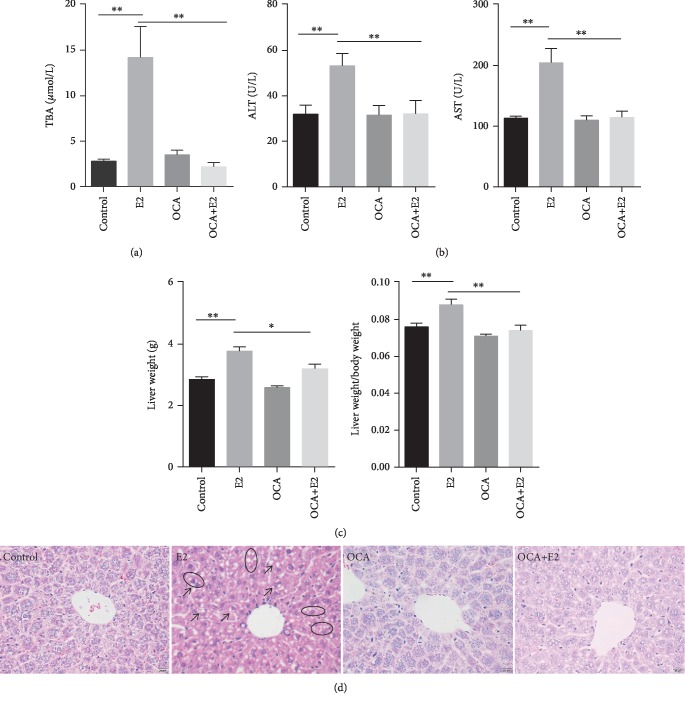
OCA pretreatment alleviated E2-induced cholestasis. All pregnant mice except controls were s.c. injected with E2 (0.625 mg/kg) once daily from GD13 to GD17. In the OCA+E2 groups, pregnant mice were administered with OCA (5 mg/kg) by gavage once daily from GD12 to GD17. All dams were sacrificed on GD18. (a) Serum total bile acid levels. (b) Serum ALT and AST. (c) Maternal liver weight and liver coefficient (liver weight/body weight). (d) Maternal liver sections were stained with H&E. Representative images were shown. Original magnification: ×400. Hepatic cytoplasmic organelles were loosening after E2 treatment (arrow). Hepatocyte atrophy (oval). Original magnification: ×400. Quantified data were expressed as means ± S.E.M. (*n* = 12 for each group). ^∗^*P* < 0.05, ^∗∗^*P* < 0.01.

The effects of 17*α*-ethynylestradiol (E2) on gestational cholestasis were analyzed. As expected, the levels of serum TBA, ALT, and AST were significantly higher in the E2 group than in the control group (Figures 2(a) and 2(b)). Maternal liver weight and liver coefficient were significantly increased in E2-treated mice (Figure 2(c)). Moreover, necrosis of hepatocytes, irregular arrangement of hepatic cords, and cytoplasm rarefaction were observed in E2-treated mice (Figure 2(d)). The effects of OCA on E2-induced gestational cholestasis were analyzed. Interestingly, OCA almost completely suppressed E2-induced elevation of serum TBA, ALT, and AST levels (Figures 2(a) and 2(b)). Maternal liver weight and liver coefficient were significantly lower in the OCA+E2 group as compared with the E2 group (Figure 2(c)). Additionally, OCA markedly attenuated E2-induced necrosis of hepatocytes, irregular arrangement of hepatic cords, and cytoplasm rarefaction (Figure 2(d)).

### 3.3. OCA Alleviated Fetal Intrauterine Growth Restriction during Gestational Cholestasis

**Figure 3:**
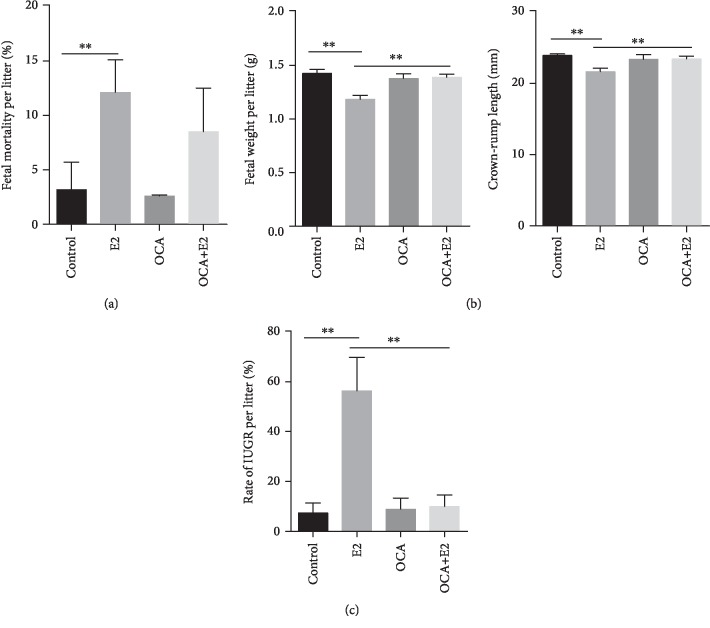
OCA pretreatment alleviated fetal intrauterine growth restriction during E2-induced cholestasis. All pregnant mice except controls were s.c. injected with E2 (0.625 mg/kg) once daily from GD13 to GD17. In the OCA+E2 groups, pregnant mice were administered with OCA (5 mg/kg) by gavage once daily from GD12 to GD17. All dams were sacrificed on GD18. (a) Fetal mortality per litter. (b) Fetal weight per litter and fetal crown-rump length per litter. (c) Rate of IUGR per litter. All data were expressed as means ± S.E.M. (*n* = 12 for each group). ^∗∗^*P* < 0.01.

The feed consumption and body weight gains of pregnant mice were measured. There were no significant differences on food consumption and body weight gains of pregnant mice among the four groups (data not shown). Fetal outcomes were presented in Table 2. No dams died throughout the pregnancy. All pregnant mice completed pregnancy. Although there were no significant differences on resorptions and live fetuses per litter among the four groups, the number of stillbirths was increased in the E2 group (Table 2). As shown in Figure 3(a), E2 treatment elevated the fetal mortality. Fetal weight and crown-rump length were subsequently analyzed. As shown in Figure 3(b), fetal weight and crown-rump length were significantly reduced in E2-treated mice. The rate of IUGR was calculated among different groups. As shown in Figure 3(c), the rate of IUGR was significantly increased in the E2 group as compared with the control group. The effects of OCA on E2-induced IUGR were analyzed. OCA significantly alleviated E2-induced reduction of fetal weight and crown-rump length (Figure 3(b)). Moreover, OCA almost completely inhibited E2-induced IUGR (Figure 3(c)).

### 3.4. OCA Alleviated the Impairments of Placental Development and Function during Gestational Cholestasis

**Figure 4:**
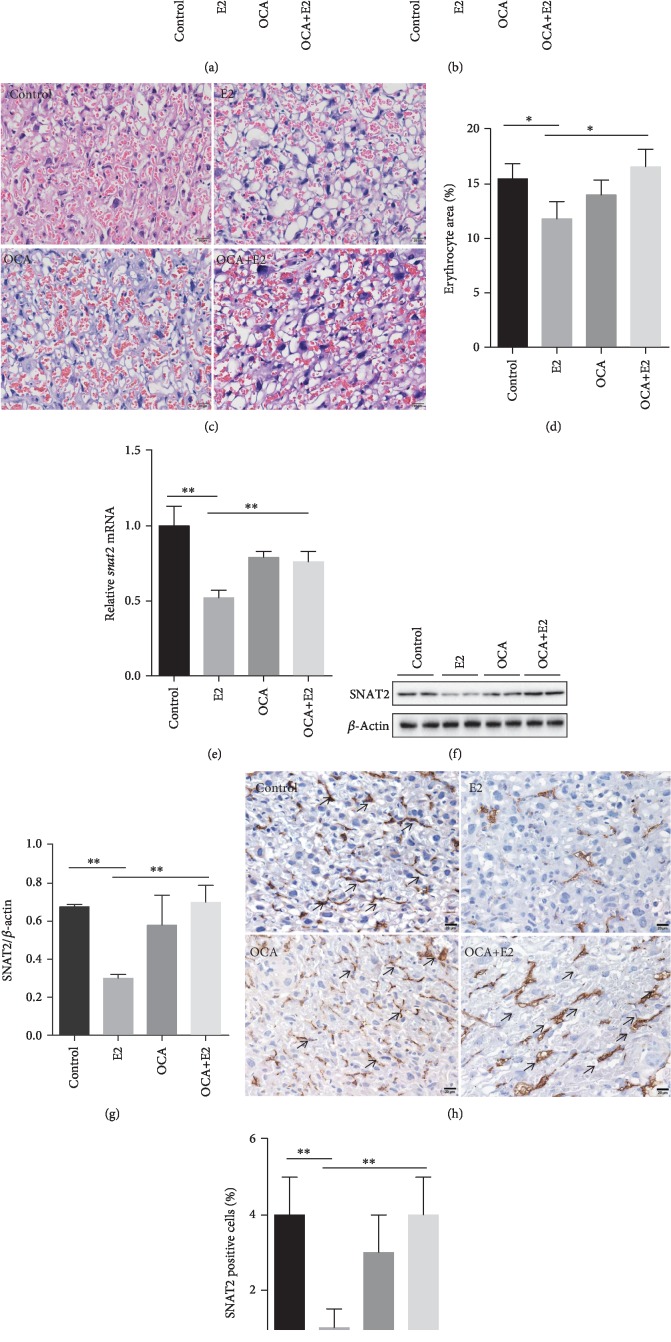
OCA pretreatment alleviated the impairments of placental development and function during E2-induced cholestasis. All pregnant mice except controls were s.c. injected with E2 (0.625 mg/kg) once daily from GD13 to GD17. In the OCA+E2 groups, pregnant mice were administered with OCA (5 mg/kg) by gavage once daily from GD12 to GD17. All dams were sacrificed on GD18. (a) Average placental weight. (b) Placental efficiency (fetal weight/placental weight). (c) Placental sections were stained with H&E. Representative images were shown. Original magnification: ×400. (d) Vascular area in the labyrinthine region was estimated from two nonconsecutive sections in each placenta using the public domain NIH ImageJ Program. All data were expressed as means ± S.E.M. (*n* = 12 for each group). ^∗∗^*P* < 0.01. (e) Placental *Snat2* mRNA was measured using real-time RT-PCR. All data were expressed as means ± S.E.M. of six samples from six different pregnant mice. (f, g) Placental Snat2 was measured using immunoblot. (f) Representative gels were shown. (g) SNAT2/*β*-actin. (h, i) SNAT2 was analyzed using IHC. (h) Representative photomicrographs were shown. Original magnification: ×400. Snat2 was observed in the labyrinth zone (arrow). (i) Snat2-positive cells were analyzed. All data were expressed as means ± S.E.M. of six samples from six different pregnant mice. ^∗∗^*P* < 0.01.

The placental development and dysfunction among the four groups were analyzed. As shown in Figure 4(a), there was a downtrend on placental weight in E2-treated mice. Additionally, placental efficiency (fetal weight/placental weight) was significantly decreased in the E2 group as compared with the control group (Figure 4(b)). Interestingly, OCA alleviated the decrease of placental efficiency (Figure 4(b)). A computerized morphometry (the public domain NIH ImageJ Program) was used to analyze cross-sectional areas of blood sinusoids in placental labyrinthine region. As shown in Figures 4(c) and 4(d), blood sinusoid area was reduced in E2-treated mice. OCA completely attenuated E2-induced reduction of blood sinusoid area in the placental labyrinth layer (Figures 4(c) and 4(d)). The effects of gestational cholestasis on placental sodium-dependent neutral amino acid transporter 2 (SNAT2) were analyzed. The levels of placental *Snat2* mRNA and SNAT2 protein were decreased during E2-induced gestational cholestasis (Figures 4(e)–4(g)). Immunohistochemistry showed that a strong SNAT2 immunoreactivity was observed in the placental labyrinth cells (Figures 4(h) and 4(i)). The effects of OCA on gestational cholestasis-downregulated placental SNAT2 were then analyzed. Interestingly, OCA pretreatment significantly attenuated E2-induced downregulation of placental *Snat2* mRNA and SNAT2 protein (Figures 4(e)–4(i)).

### 3.5. OCA Pretreatment Alleviated Oxidative Stress and Protein Nitration during E2-Induced Cholestasis

**Figure 5:**
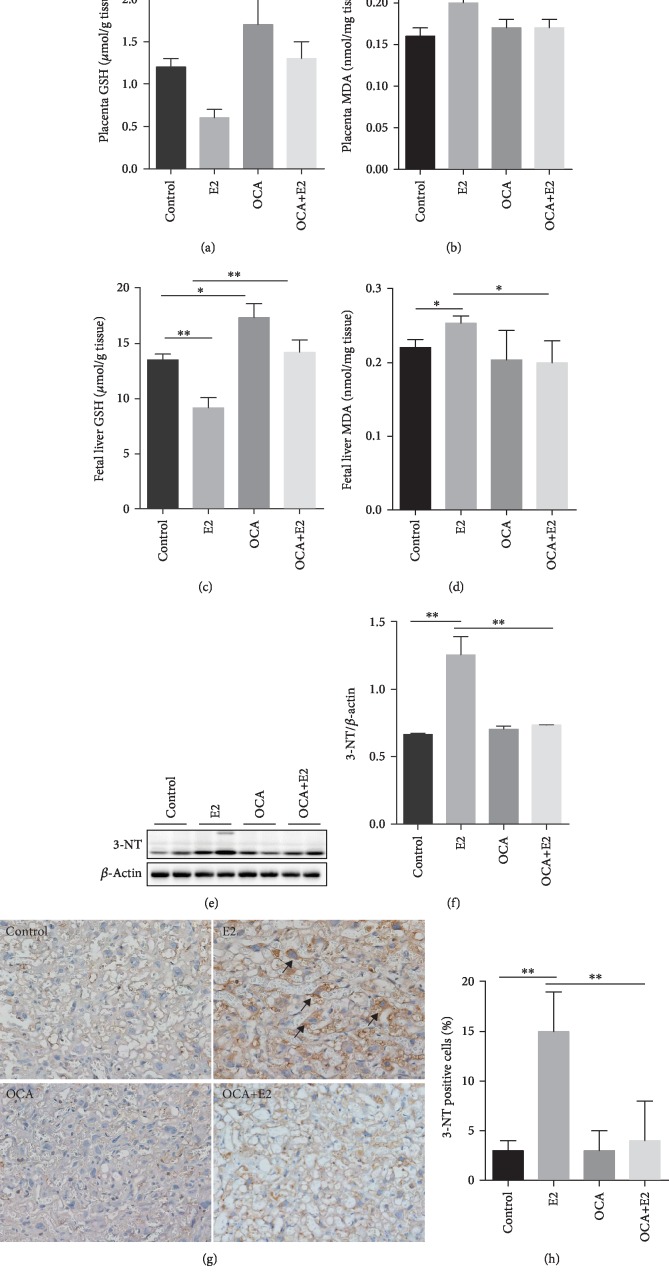
OCA pretreatment alleviated oxidative stress and protein nitration during E2-induced cholestasis. All pregnant mice except controls were s.c. injected with E2 (0.625 mg/kg) once daily from GD13 to GD17. In the OCA+E2 groups, pregnant mice were administered with OCA (5 mg/kg) by gavage once daily from GD12 to GD17. All dams were sacrificed on GD18. (a) Placental GSH level (*n* = 12 for each group); (b) placental MDA content (*n* = 12 for each group). (c) Fetal liver GSH level (*n* = 12 for each group); (d) fetal liver MDA content (*n* = 12 for each group). (e, f) Placental 3-NT residues were analyzed using immunoblot. (e) Representative gels were shown. (f) 3-NT/*β*-actin (*n* = 6 for each group). (g, h) Placental 3-NT residues were analyzed by IHC. (g) Representative photomicrographs were shown. Original magnification: ×400. Placental 3-NT residues were mainly distributed in mononuclear sinusoidal TGCs of the labyrinth zone (arrow). (h) The percentages of 3-NT^+^ cells were evaluated among different groups (*n* = 6 for each group). ^∗^*P* < 0.05, ^∗∗^*P* < 0.01.

The effects of OCA on GSH depletion and lipid peroxidation in placenta and fetal liver during E2-induced cholestasis were analyzed. Decreased degree of GSH was observed in the placenta and fetal liver from the E2 group (Figures 5(a) and 5(c)), indicating enhanced oxidative damage. By contrast, the level of MDA in placenta and fetal liver, a marker of lipid peroxidation, was elevated in the E2 group (Figures 5(b) and 5(d)). Interestingly, OCA pretreatment significantly attenuated E2-induced GSH depletion and lipid peroxidation in placenta and fetal liver (Figures 5(a)–5(d)). The effects of OCA on protein nitration during E2-induced cholestasis were then analyzed. As expected, placental 3-NT residue, a marker of protein nitration, was highly increased in E2-treated mice (Figures 5(e) and 5(f)). Immunohistochemistry showed that 3-NT residues were mainly distributed in mononuclear sinusoidal trophoblast giant cells (TGCs) of the labyrinth zone in E2-treated mice (Figures 5(g) and 5(h)). Of interest, OCA significantly alleviated E2-induced protein nitration in the placentas (Figures 5(e)–5(h)).

### 3.6. OCA Inhibited the Upregulation of Placental NADPH Oxidase-4 and Antioxidant Genes during E2-Induced Cholestasis

**Figure 6:**
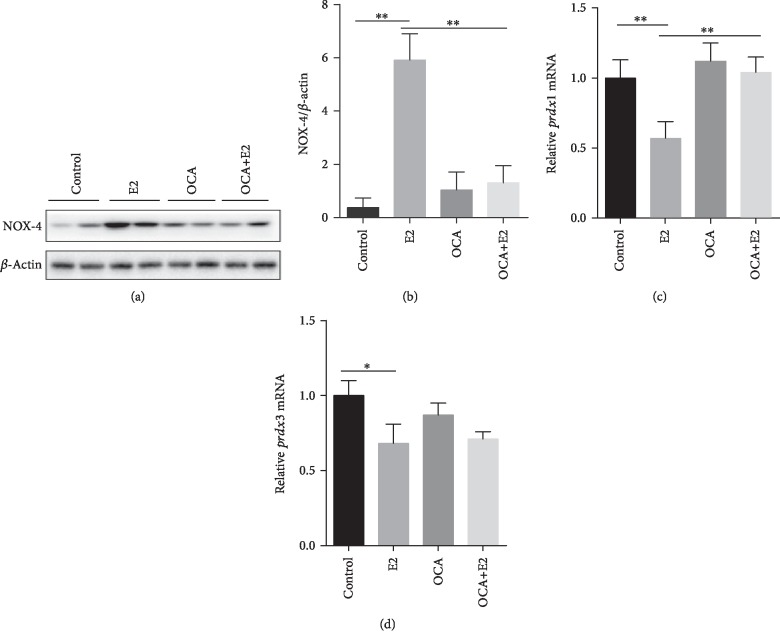
OCA inhibited the upregulation of placental NADPH oxidase-4 and antioxidant genes during E2-induced cholestasis. All pregnant mice except controls were s.c. injected with E2 (0.625 mg/kg) once daily from GD13 to GD17. In the OCA+E2 groups, pregnant mice were administered with OCA (5 mg/kg) by gavage once daily from GD12 to GD17. All dams were sacrificed on GD18. (a, b) Placental NADPH oxidase 4 (NOX4) was measured using immunoblot. (a) Representative gels were shown. (b) NOX4/*β*-actin. (c, d) The expressions of placental peroxiredoxins (*prdx*) were measured using real-time RT-PCR: (c) *prdx1* mRNA and (d) *prdx3* mRNA. Quantified data were expressed as means ± S.E.M. of six samples from six different pregnant mice. ^∗^*P* < 0.05, ^∗∗^*P* < 0.01.

The protein level of NADPH oxidase 4 (NOX4) was measured using western blotting. As shown in Figures 6(a) and 6(b), the protein level of placental NOX-4 was significantly increased in pregnant mice of E2-induced cholestasis. Interestingly, OCA significantly inhibited E2-induced increase of NOX-4 protein (Figures 6(a) and 6(b)). Placental peroxiredoxin (*prdx*) 1 and *prdx3* genes, two antioxidant genes, were measured using real-time RT-PCR. As shown in Figures 6(c) and 6(d), E2 significantly downregulated gene expressions of *prdx*1 and *prdx3* in the placentas. Additionally, OCA pretreatment inhibited E2-induced downregulation of placental *prdx1* gene expression (Figures 6(c) and 6(d)).

### 3.7. OCA Upregulated Placental Nrf2 Signaling during Gestational Cholestasis

**Figure 7:**
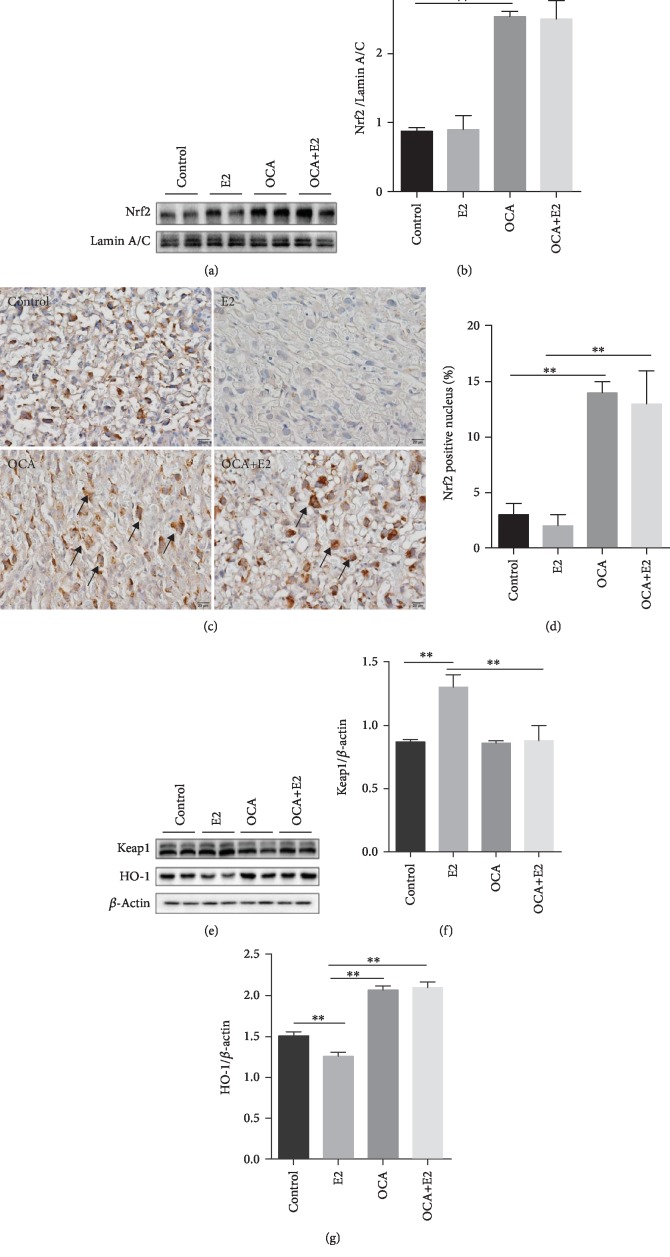
OCA pretreatment upregulated placental Nrf2 signaling during E2-induced cholestasis. All pregnant mice except controls were s.c. injected with E2 (0.625 mg/kg) once daily from GD13 to GD17. In the OCA+E2 groups, pregnant mice were administered with OCA (5 mg/kg) by gavage once daily from GD12 to GD17. All dams were sacrificed on GD18. (a, b) Placental Nrf2 was measured using immunoblot. (a) Representative gels were shown. (b) Nrf2/Lamin A/C. (c, d) Placental Nrf2 was analyzed using IHC. (c) Representative photomicrographs were shown. Original magnification: ×400. Placental mononuclear sinusoidal TGCs were stained strong positive (arrows). (d) The percentages of Nrf2-positive nuclei were evaluated among different groups. (e–g) Placental Keap1 and HO-1 were measured using immunoblot. (e) Representative gels were shown. (f) Keap1/*β*-actin; (g) HO-1/*β*-actin. Quantified data were expressed as means ± S.E.M. of six samples from six different pregnant mice. ^∗∗^*P* < 0.01.

The effects of OCA on placental Nrf2 signaling pathway were analyzed. As shown in Figures 7(a) and 7(b), the nuclear protein level of placental Nrf2 was significantly increased in OCA-treated and OCA+E2-treated mice. Immunohistochemistry showed that a strong nuclear Nrf2 immunoreactivity was detected in mononuclear sinusoidal TGCs of the placental labyrinth zone in OCA-treated and OCA+E2-treated mice (Figures 7(c) and 7(d)). Moreover, placental Keap1, an inhibitory protein of Nrf2, was significantly increased in E2-treated mice, whereas it was significantly decreased in OCA+E2-treated mice (Figures 7(e) and 7(f)). Placental HO-1, a Nrf2 downstream protein, was measured using western blotting. As shown in Figures 7(e) and 7(g), the protein level of placental HO-1 was markedly downregulated in E2-treated mice, whereas it was significantly upregulated in OCA-treated and OCA+E2-treated mice (Figures 7(e) and 7(g)).

## 4. Discussion

The present study investigated the effect of OCA pretreatment on fetal death and IUGR during E2-induced gestational cholestasis in mice. As expected, treatment with E2 during late pregnancy induced gestational cholestasis in mice. Our results demonstrated that gestational cholestasis induced by E2 increased fetal mortality. Moreover, gestational cholestasis caused reduction of fetal weight and crown-rump length. Gestational cholestasis elevated the incidence of fetal IUGR in mice. OCA alone had no effect on pregnancy outcomes, but OCA pretreatment significantly attenuated the reduction of fetal weight and crown-rump length during E2-induced gestational cholestasis. Additionally, OCA pretreatment decreased the incidence of fetal IUGR during E2-induced gestational cholestasis in mice.

The placentas play an essential role in the development of the fetus. Increasing evidence demonstrated that placental insufficiency was one of the major factors for fetal death and IUGR [35–37]. Indeed, several case-control studies found that gestational cholestasis was associated with morphological abnormalities in the placenta, such as vasoconstriction of chorionic veins, smaller blood vessels, reduced intervillous space, and increased occurrence of syncytial knots [38–40]. In the present study, a downward trend on placental weight was observed in pregnant mice with cholestasis. The labyrinth layer in the placentas, the site of oxygen and nutrient exchange between the mother and the fetus, was severely disrupted, with a reduction in the internal space of fetal and maternal blood vessels. Sodium-coupled neutral amino acid transporter- (SNAT-) 2 is a membrane transport protein and highly expressed in rodent and human placentas. The function of the placental SNAT-2 is to transport neutral amino acids from the mother to the fetus [41]. A recent case-control study showed that placental SNAT-2 protein level was significantly lower in the IUGR fetus than in the appropriately grown for gestational age fetus [42]. Animal experiments indicated that the downregulation of placental SNAT-2 expression was one of the key factors responsible for fetal IUGR [43]. The present study found that SNAT-2 protein and gene in the placentas was downregulated in pregnant mice with cholestasis, highlighting that gestational cholestasis impairs not only placental development but also placental function. Of interest, OCA pretreatment attenuated the reduction of blood sinusoid area in the placental labyrinth layer and inhibited the downregulation of placental SNAT2 during E2-induced gestational cholestasis. Taken together, these results suggest that OCA pretreatment protects against IUGR during E2-induced gestational cholestasis which may be through alleviating impairments of placental development and function.

Excess reactive nitrogen species (RNS) and reactive oxygen species (ROS) productions are involved in cholestasis-induced liver injury and placental impairment [22, 23, 44]. Excess RNS and ROS productions were also associated with fetal death and IUGR [45, 46]. Several studies found that FXR has an antioxidant effect [28, 29]. The present study showed that OCA pretreatment inhibited GSH depletion and lipid peroxidation in placenta and fetal liver during E2-induced gestational cholestasis. In addition, OCA pretreatment significantly suppressed protein nitration in mononuclear sinusoidal trophoblast giant cells of placental labyrinth layer during E2-induced gestational cholestasis. Numerous data showed that NADPH oxidases were one of the main sources of cellular ROS production [47]. On the other hand, peroxiredoxins (Prdx) belong to ubiquitous family of antioxidant enzymes, which are known to scavenge RNS and ROS [48, 49]. The present study found that the protein level of NADPH oxidase 4 (NOX4) in placenta was significantly increased in pregnant mice of E2-induced cholestasis. Additionally, placental *prdx1* and *prdx3* were downregulated in pregnant mice of E2-induced cholestasis. Interestingly, OCA pretreatment obviously repressed the increase of placental NOX4 during E2-induced gestational cholestasis. Moreover, OCA pretreatment significantly suppressed downregulation of prdx1 gene. The present results indicate that OCA pretreatment alleviates placental damage at least partially through regulating placental NADPH oxidases and antioxidant genes.

Nuclear factor E2-related factor 2 (Nrf2)/Kelch-like ECH-associated protein 1 (Keap1) is one of the master antioxidant response element signaling pathways [50]. Nrf2 is activated through inactivation of Keap1. Subsequently, Nrf2 escapes from the ubiquitination system and then gradually transfers into the nucleus, where it binds to antioxidant response elements (AREs) in the promoter regions of its target genes, such as heme oxygenase-1 (HO-1) [51]. Numerous reports demonstrated that Nrf2/Keap1 pathway activation played an important role in alleviating placental damage through inhibiting oxidative stress [52, 53]. A recent study found that weight and both total and labyrinth layer volume were significantly reduced in the placenta of Nrf2 knockout mice, highlighting deficiency in Nrf2 signaling impair placental development and dysfunction [54]. The present study found that the protein level of Keap1 was downregulated in OCA-treated pregnant mice, whereas the protein level of nuclear Nrf2 was upregulated in OCA-treated pregnant mice. In addition, HO-1, a target gene of Nrf2 pathway, is also upregulated in OCA-treated pregnant mice. These results suggest that OCA pretreatment alleviates placental damage may be partially through activating placental Nrf2/Keap1 pathway.

Bile acid accumulation is the major pathological characteristic of cholestasis. An epidemiological study reported that maternal serum TBA levels at diagnosis and at delivery were correlated positively with umbilical cord blood TBA levels, which provides evidence that bile acids can be transported across the placenta [55]. Recently, several reports found that bile acids induced oncosis, necrotic cell death, and apoptosis, suggesting the direct toxic effect of bile acids [56, 57]. The present study showed that serum TBA level was obviously increased in pregnant mice with cholestasis. Moreover, placental, maternal, and fetal hepatic FXR signaling was upregulated in OCA-treated pregnant mice. OCA pretreatment almost completely depressed the increase of serum TBA level in pregnant mice of E2-induced cholestasis. FXR is expressed at low levels in placenta [29]. But it is not obvious in our immunoblot results. In fact, this is due to the placental protein loading in the lane which is higher than that in the liver in our immunoblot. Thus, the present study does not exclude the fact that OCA pretreatment alleviated cholestasis-associated fetal IUGR due to the reduction of bile acid levels.

The present study has several flaws. First, our study had not investigated the effects of posttreatment with OCA on gestational cholestasis-induced fetal IUGR. In view of the model of gestational cholestasis, our data suggest that OCA is a potential pharmacological agent to treat cholestasis and oxidative stress-associated adverse pregnancy outcomes. Second, our study only measured placental SNAT2 mRNA and protein for evaluating placental amino acid transport capacity. More tests should be performed to fully assess placental functions.

In summary, our study investigated the effect of OCA pretreatment on IUGR during E2-induced gestational cholestasis in mice. We found that gestational cholestasis induced by E2 attenuated IUGR. OCA pretreatment protected against gestational cholestasis induced by E2-caused IUGR through suppressing placental oxidative stress and maintaining bile acid homeostasis. Overall, the present study provides evidence for OCA as potential pharmacological agents to prevent gestational cholestasis and oxidative stress-associated adverse pregnancy outcomes.

## Figures and Tables

**Table 1:** Primers for real-time RT-PCR.

Genes	Sequence	Length
*18S*	Forward: 5′-GTAACCCGTTGAACCCCATT-3′	151
	Reverse: 5′-CCATCCAATCGGTAGTAGCG-3′	
*Cyp7a1*	Forward: 5′-ACTAGGGAAGTTTCGACATGC-3′	162
	Reverse: 5′-ATGGTGTGGTTCTTGGAGGT-3′	
*Cyp8b1*	Forward: 5′-TTGCAAATGCTGCCTCAACC-3′	118
	Reverse: 5′-TAACAGTCGCACACATGGCT-3′	
*Bsep*	Forward: 5′-GACTTTCCACAGTGGCGTCT-3′	123
	Reverse: 5′-AGTGTCAGTGGCCTTTCGTA-3′	
*Mdr2*	Forward: 5′-GATGGATCTTGAGGCAGCGA-3′	191
	Reverse: 5′-GAGCTATGGCCATGAGGGTG-3′	
*Mrp2*	Forward: 5′-CTTCCCTTGAGGCAGATGGT-3′	189
	Reverse: 5′-CCCAAGGGAATCCACACAAGA-3′	
*Snat2*	Forward: 5′-ACCTCACCTGCTCGTCAAAG-3′	117
	Reverse: 5′-TGGTTGTCATGGCACCTCTC-3′	
*Prdx1*	Forward: 5′-GTCCCACGGAGATCATTGCT-3′	110
	Reverse: 5′-AGAATCCACAGAAGCGCCAA-3′	
*Prdx3*	Forward: 5′-TCGGTATCTCCGCCTATCGT-3′	110
	Reverse: 5′-GAGGACCAGAGCAACCTTCC-3′	

**Table 2:** Fetal outcomes among different groups.

Parameters	Control	E2	OCA	OCA+E2
Number of pregnant mice (*n*)	12	12	12	12
Litters of preterm delivery (*n*)	0	0	0	0
Resorptions per litter (*n*)	0.3 ± 0.2	0.3 ± 0.2	0.2 ± 0.1	0.2 ± 0.2
Live fetuses per litter (*n*)	12.8 ± 1.1	10.3 ± 0.9	13.1 ± 1.4	12.9 ± 1.0
Dead fetuses per litter (*n*)	0.3 ± 0.2	1.4 ± 0.5^†^	0.1 ± 0.1	0.8 ± 0.4
Placental diameter (mm)	8.31 ± 0.13	8.30 ± 0.12	8.36 ± 0.15	8.43 ± 0.39

^†^ *P* < 0.05.

## Data Availability

All the experimental data used to support the findings of this study are included within the article.

## Abbreviations

OCA: Obeticholic acid
IUGR: Intrauterine growth restriction
ICP: Intrahepatic cholestasis of pregnancy
UDCA: Ursodeoxycholic acid
FXR: Farnesoid X receptor
E2: 17*α*-Ethynylestradiol
GD: Gestational day
TBA: Total bile acid
ALT: Alanine aminotransferase
AST: Aspartate aminotransferase
Cyp7a1: Cholesterol 7a-hydroxylase
Cyp8b1: Oxysterol 12b-hydroxylase
Bsep: Bile salt export pump
Mdr: Multidrug resistance protein
Mrp: Multidrug resistance-associated protein
GSH: Glutathione
MDA: Malondialdehyde
SNAT2: Amino acid transporter 2
TGCs: Trophoblast giant cells
NOX4: NADPH oxidase 4
prdx: Peroxiredoxin
Nrf2: NF-E2-related factor 2
RNS: Reactive nitrogen species
ROS: Reactive oxygen species
IHC: Immunohistochemistry.
